# Is C-reactive protein sufficient to guide antimicrobial therapy for lower respiratory tract infections among children? Results from a stepped-wedge cluster randomized trial in Uganda

**DOI:** 10.1371/journal.pmed.1004467

**Published:** 2024-09-17

**Authors:** Chris A. Rees, Prashant Mahajan, Quique Bassat

**Affiliations:** 1 Division of Pediatric Emergency Medicine, Emory University School of Medicine, Atlanta, Georgia, United States of America; 2 Department of Pediatrics, University of Michigan, Ann Arbor, Michigan, United States of America; 3 Department of Emergency Medicine, University of Michigan, Ann Arbor, Michigan, United States of America; 4 Centro de Investigação em Saúde de Manhiça [CISM], Maputo, Mozambique; 5 ISGlobal—Hospital Clínic, Universitat de Barcelona, Barcelona, Spain; 6 ICREA, Barcelona, Spain; 7 Pediatrics Department, Hospital Sant Joan de Déu, Universitat de Barcelona, Esplugues, Barcelona, Spain; 8 CIBER de Epidemiología y Salud Pública, Instituto de Salud Carlos III, Madrid, Spain

The accurate identification of young children with non-severe lower respiratory tract infections who may benefit from antimicrobial therapy is crucial both for individual patient management but also to curb crowing rates of antimicrobial resistance that pose a public health threat. Research by Ciccone and colleagues suggests that using a C-reactive protein guided approach to the evaluation of young children with lower respiratory tract infections may help reduce antimicrobial prescription without increasing rates of treatment failure.

Despite more than a 75% global reduction in fatal cases of lower respiratory tract infections (LRTI) from the 1990s to the 2020s, LRTIs continue to be the leading individual cause of morbidity and mortality for children aged 2 to 59 months [1]. Despite such significant burden, there is no clinically feasible reference standard for the diagnosis of LRTI. A definitive diagnosis of LRTI can only be made through lung biopsy, which is neither feasible nor necessary in routine clinical practice. Due to the assumption that a high proportion of fatal LRTIs are bacterial in nature, the World Health Organization (WHO) recommends that the diagnosis and treatment of LRTI in low- and middle-income countries be driven by severity and clinical presentation, with more severe cases requiring referral to the hospital. Specifically, the WHO recommends that children aged between 2 and 59 months with a cough and age-adjusted tachypnea be treated with antibiotics either in the community for non-severe cases or in the hospital for severe cases [2]. WHO-defined LRTI does not require radiographic confirmation. However, prior studies have questioned the accuracy of clinical signs and symptoms for WHO-defined LRTI compared to radiographic pneumonia [3].

It should be noted, however, that chest radiographic findings cannot be used to accurately distinguish between viral and bacterial etiologies [4]. To add further complexity, findings from a multi-center study involving extensive etiological testing suggest that as many as 60% of young children with LRTIs had viruses (and not bacterial infections), and thus may not directly benefit from antimicrobial therapy [5]. This may explain why findings from randomized controlled trials suggest that children with non-severe WHO-defined LRTI treated with shorter courses of antimicrobial therapy (i.e., 3 days) [6] and even with placebo [7] had no difference in rates of treatment failure or symptom relapse compared to children with non-severe WHO-defined LRTI treated with 5 and 3 days of antimicrobial therapy, respectively. Moreover, several studies have demonstrated that the indiscriminate administration of antimicrobial therapy to children can contribute to antimicrobial resistance [8]. Therefore, it is imperative that antimicrobial therapy be tailored to the child with high risk of a bacterial pathogen and potentially avoided if a child has low risk of a bacterial infection. Thus, the crucial question in the management of LRTI in children in low- and middle-income countries is: Which child requires antibiotics and which child can be safely managed expectantly without antibiotics?

The study published by Ciccone and colleagues aims to answer this question [9]. The authors conducted a stepped-wedge cluster randomized trial in 15 villages in Uganda that incorporated the use of point-of-care C-reactive protein (CRP) levels from children aged 2 to 59 months collected by lay community health workers, with the primary outcome of antimicrobial prescription reduction for WHO-defined LRTI. They analyzed data on 1,220 children who met the study’s inclusion criteria (i.e., children aged 2 to 59 months evaluated by a community health worker, with fever [>38°C] or subjective fever within the past 7 days, and tachypnea [respiratory rate >30 breaths per minute] or cough). Community health workers used a predetermined CRP level of ≥40 mg/L to determine if a child needed the prescription of a five-day course of oral amoxicillin. For children with a CRP of <40 mg/L, community health workers were instructed to recommend adequate hydration and antipyretics as needed for fever.

The investigators observed a nearly 25% reduction in the prescription of antimicrobial therapy for suspected LRTI among enrolled participants. Importantly, the investigators did not identify differences in their secondary outcomes that described potential negative effects from withholding antimicrobial prescriptions. The authors concluded that the incorporation of CRP into the routine management of LRTI among young children has the potential to reduce antimicrobial therapy administration with no difference in clinical outcomes.

This well-designed trial has many strengths. The investigators used a pragmatic design and trained community health workers, who are often front-line providers for young children with LRTI in low- and middle-income countries. The authors note that the number needed to test to avoid one course of amoxicillin was four. This is noteworthy as the number needed to treat for common medications such as acetaminophen for fever is as high as seven [10]. Furthermore, although a formal cost benefit analysis was not conducted, the authors note that the associated cost of testing 4 individuals to reduce one course of antibiotics would be roughly $6.80 USD. Of course, this is much higher than the reported cost of one course of amoxicillin (i.e., $0.50 to $1.30), but the larger public health benefits from improved antimicrobial stewardship are difficult to measure.

Despite these strengths, there are additional areas of opportunity that this study presents. The authors chose a CRP level of ≥40 mg/L to guide the community health worker’s decision to provide antimicrobial therapy or not. The authors report that the rationale for this decision was based on prior studies that suggest that withholding antimicrobial therapy in children with a CRP of <40 mg/L was safe, and that level also had high negative predictive value for radiographic pneumonia. Ideally, biomarker levels used for clinical decision-making should be empirically derived against the outcome of interest. Thus, further studies are needed to elucidate a CRP level at which antimicrobial therapy can be withheld without negative outcomes for children with LRTI. Next, children enrolled in this study had suspected LRTI, which is diagnosed clinically per current WHO recommendations with no need for blood draws. If CRP were to be integrated into routine practice, the potential inconveniences to children who would otherwise not have a blood draw and for the health care providers responsible for the blood draws needs to be considered. Another area for opportunity in future investigations is the inclusion of pulse oximetry. Prior studies have demonstrated that hypoxia has been associated with poor outcomes in children with LRTI [11]. However, nearly 80% of participants had missing data on pulse oximetry in this study. Future studies may consider the inclusion of both pulse oximetry and diagnostic, but also prognostic, biomarkers to more accurately identify young children with suspected LRTI at risk of severe outcomes [12], and/or in need of antimicrobial therapy and referral to hospital settings. Furthermore, additional studies are ongoing to assess the ability of point-of-care lung ultrasound to accurately identify young children with WHO-defined LRTI without abnormal findings who may not benefit from antimicrobial therapy. Lastly, it is important to note that this study was limited to children with mild LRTI. Withholding antimicrobial therapy from a child with suspected LRTI in severe respiratory distress, regardless of their CRP level, is unwise without further risk stratification and guidance.

The incorporation of CRP into the approach community health workers take to prescribe amoxicillin to young children with suspected LRTI is a step toward safe and improved antimicrobial stewardship. However, just as WHO guidelines have balanced feasibility and generalizability for LRTI management, similar considerations will be required before CRP can be integrated into routine clinical practice.
